# Mesenchymal stem cells for sensorineural hearing loss: protocol for a systematic review of preclinical studies

**DOI:** 10.1186/s13643-019-1015-7

**Published:** 2019-05-25

**Authors:** Kevin T. Chorath, Matthew J. Willis, Nicolas Morton-Gonzaba, Walter J. Humann, Alvaro Moreira

**Affiliations:** 1 Department of Pediatrics, Division of Neonatology, The University of Texas Health San Antonio, 7300 Floyd Curl Dr. MC-7812, San Antonio, TX 78229 USA; 2 Department of Otolaryngology, University of Tennessee Health Science Center, Memphis, TN USA

**Keywords:** Sensorineural hearing loss, Preclinical, Systematic reviews, Risk of bias, Stem cells, Mesenchymal stem cells, Animal models

## Abstract

**Background:**

Sensorineural hearing loss (SNHL) is the most common form of hearing impairment and is characterized by a loss of receptor hair cells and/or spiral ganglion neurons. Regenerative stem cell therapy could potentially restore normal hearing and slow the progression of hearing loss in patients. Preclinical animal studies have demonstrated that mesenchymal stem cells (MSCs) could be a promising new therapy for this condition. These findings have prompted investigators to begin human clinical trials to assess the safety and efficacy of MSCs for the treatment of SNHL. The objective of the proposed systematic review is to examine the efficacy of MSCs as a therapy for SNHL in animal models.

**Methods:**

We will include preclinical animal studies of SNHL in which MSCs are administered, and outcomes are compared against MSC-naïve controls. The primary outcome will include audiologic tests that are routinely used in experimental studies of hearing loss, such as auditory brainstem response (ABR) and distortion product otoacoustic emissions testing (DPOAE). Secondary outcomes will include histology, microscopy, gene protein expression, and behavioral responses of animals. Electronic searches of MEDLINE via PubMed, Scopus, ScienceDirect, and Cumulative Index to Nursing and Allied Health Literature (CINAHL) will be performed. Search results will be screened independently and in duplicate. Data from eligible studies will be extracted, pooled, and analyzed using random effects models. Risk of bias and publication bias will be assessed using the Systematic Review Center for Laboratory Animal Experimentation (SYRCLE) risk of bias tool and Funnel Plots/Egger’s regression tests, respectively.

**Discussion:**

This systematic review will provide a summary of the efficacy of MSC therapy in animal models of SNHL, utilizing functional hearing assessment as a primary outcome. Findings from this review are important because they can elucidate research gaps that should be addressed in future preclinical studies and in turn can be translated into clinical studies.

**Systematic review registration:**

CAMARADES (http://www.dcn.ed.ac.uk/camarades/)

**Electronic supplementary material:**

The online version of this article (10.1186/s13643-019-1015-7) contains supplementary material, which is available to authorized users.

## Background

Sensorineural hearing loss (SNHL) is the most common form of hearing impairment [1]. SNHL affects up to 4.6 infants per 1000 live births [1] and has a multifactorial etiology, including congenital anomalies, exposure to ototoxic drugs, noise induced, and/or aging [2]. Individuals with any degree of SNHL are at increased risk for language delay, depression, and cognitive decline [3, 4].

Current approaches to treat SNHL focus on the use of hearing aids and cochlear implants. These devices bypass the damaged ear by augmenting sound intensity to a detectable threshold [5]. Although supportive, these interventions are not curative. Future therapies should focus on restoring and/or attenuating progressive hearing loss.

Major advancements in regenerative medicine have stimulated interest in the potential of cell-based therapies for SNHL. Mesenchymal stem/stromal cells (MSCs) have emerged as the cell line with most therapeutic potential due to their ease for isolation, regenerative properties, and easy and non-invasive retrieval [6]. Preclinical studies have now demonstrated that MSCs provide favorable results in orthopedics, myocardial infarctions, liver fibrosis, respiratory distress syndrome, and autoimmune conditions [7–10]. MSCs are novel and viable options to treat SNHL since they have the potential to differentiate into hair cells and spiral ganglion neurons. As well, preclinical animal studies have revealed an improvement in auditory functioning [2, 11–13]. Currently, a phase I/II clinical trial (NCT02038972) is evaluating the safety and efficacy of umbilical cord-derived MSCs for treatment of SNHL. Despite these advancements, there has been no systematic synthesis of preclinical studies evaluating the use of MSCs for SNHL.

The objective of the proposed systematic review is to examine the efficacy of MSCs as a therapy for SNHL in animal models. Results from this work may help guide subsequent preclinical studies, as well as potentially impact key elements in future clinical trials.

### Study question

In preclinical studies of SNHL, does administration of MSCs improve hearing outcomes when compared to placebo or untreated controls?

## Methods/design

### Protocol and registration

The protocol was developed through discussions with our scientific research team. The team was comprised of clinicians (AM) and translational scientists (KC, MW, NM, WH). This protocol will adhere to the Systematic Review Protocol for Animal Intervention Studies guidelines set forth by the Systematic Review Centre for Laboratory Animal Experimentation (SYRCLE) [14]. This protocol has been registered under Collaborative Approach to Meta-Analyses and Review of Animal Data from Experimental Studies (CAMARADES) website, found at http://www.dcn.ed.ac.uk/camarades/research.html#protocols, and is also attached as an additional file [see Additional file 1].

### SNHL definition and type of studies

In the current study, SNHL is a type of hearing loss in which the root cause lies in the vestibulocochlear nerve or the inner ear. SNHL is characterized by a loss of receptor hair cells and/or spiral ganglion neurons, which carry afferent signals from the cochlea [12]. There are a variety of causes of SNHL, most commonly genetic dysfunction, ototoxic drugs like aminoglycosides and loop diuretics, noise exposure, and aging [11]. In preclinical studies, SNHL is induced via pharmacological agent, immunological conditioning, hereditary dysfunction, and noise induction [2, 12, 13].

The inclusion criteria are as follows: full-text original papers, controlled animal intervention studies (randomized and non-randomized) that evaluate the therapeutic efficacy of mesenchymal stem cells (MSCs) for sensorineural hearing loss in animals, regardless of methodological quality.

The exclusion criteria are as follows: review and editorial articles, non-intervention studies, no control group, human studies, exclusive in vitro work, or co-intervention studies.

### Types of preclinical animal models

We will include preclinical in vivo models of SNHL that represent the pathophysiological features of human SNHL. Eligible animal models include healthy mammals of all genders and ages. A variety of methods to induce SNHL exist, which include surgical injection of neomycin and oubain mixture into the cochlea, animals with hereditary SNHL, and β-tubulin immunization [2, 12, 13]. These models may require surgery or extensive manipulation that is subject to technical variability. However, these models provide a predictable disease phenotype and share a common feature to human SNHL: histological evidence of cochlear cell loss or elevated auditory brainstem response (ABR) thresholds.

### Intervention vs. control group

The intervention group will include animals receiving MSCs after the induction of SNHL. Studies treating animals with cell-free products (microRNA, exosomes, microvesicles, etc.) will be excluded. An MSC will be defined per the International Society for Cellular Therapy [15]. The preclinical comparison group will include animals from studies that have experimentally induced or hereditary SNHL but have not been administered an MSC, including placebo-controlled and sham-operated animals.

### Type of interventions

The intervention group will include animals that receive administration(s) of MSCs regardless of dosage, timing, delivery routes, and frequency of intervention. MSC sources can be autologous, xenogeneic, allogenic, and syngeneic. Furthermore, we will add studies that have modified (genetically, pharmacologically) MSCs and incorporate them as single or adjuvant agents (with scaffolds, other cells, or conditioned media).

### Primary/secondary endpoints

The current gold standard for the diagnosis and evaluation of sensorineural hearing loss is a functional hearing assessment. There are multiple approaches to assess functional hearing in preclinical studies; therefore, this systematic review will include studies that utilize audiologic tests that are routinely used in experimental studies of hearing loss [16]. Therefore, the primary outcomes will include:

1. Cochlear microphonic receptor potentials
2. Auditory brainstem response (ABR)
3. Electrocochleography
4. Distortion product otoacoustic emissions testing (DPOAE)
5. Summating potentials
6. Tympanometry
7. Compound action potentials
8. Brainstem auditory evoked potentials (BAEPs)

The secondary endpoints will include:

1. Histology
2. Microscopy
3. Gene protein expression
4. Behavioral responses of animals

Results from studies that incorporate the same audiologic testing will be pooled for further analysis. If the data is appropriate for quantitative synthesis, meta-analysis will be conducted using a random effects model to generate forest plots. The estimated effect of MSCs on functional hearing will be analyzed with standard mean difference (SMD) and 95% confidence intervals (CI). Statistical heterogeneity between studies will calculated using *I*^2^ metrics, and further subgroup analysis using meta-regression will be performed to assess the impact of all variables on the study effect. The variables examined will include animal type, age, sex, species, and strain; type of SNHL induction; type and tissue source of MSC; and timing, frequency, and route of cell administration.

### Timing of outcome measurements

Outcomes that meet primary +- secondary end point (before and after intervention, short- vs. long-term, and repeated studies) will be included.

### Search strategies

We will conduct a comprehensive search of MEDLINE via PubMed, Scopus, ScienceDirect, and Cumulative Index to Nursing and Allied Health Literature (CINAHL) databases. Only articles in the English language will be included in the review. There will be no restrictions to publication dates.

Search strategies will use a combination of controlled vocabulary (e.g., mesenchymal stem cells, sensorineural hearing loss, animal) and keywords (e.g., SNHL, ABR, MSC), and parsing will be formatted accordingly to each database. An additional file with the search terms utilized for PubMed is attached [see Additional file 2]. Furthermore, a manual review of references of the selected articles will be performed. A sample combination of search terms and keywords includes:

i. Mesenchymal stem cell, mesenchymal stromal cell, MSC
ii. Sensorineural hearing loss, hearing impairment, SNHL
iii. Animal, preclinical, experimental

### Study selection

Titles and abstracts of the search results will be screened independently by two individuals (KC, MW). Full-text articles will be reviewed based on the inclusion and exclusion criteria. Disagreements between individuals will be resolved by consensus or by a third member of the group (AM). Reasons for study exclusion will be recorded and presented in accordance with the SYRCLE Guidelines.

### Data collection

Data will be extracted independently by two individuals (KC, MW) using a standardized form approved by all investigators. If any data is missing or further information is required, the authors of the manuscripts will be emailed. In case of no response after a reminder email at 1 month, studies will be excluded from the analysis. Specific data elements collected for this review are listed in Table 1.

**Table 1:** Study data collection

Study characteristic	Specific items
Study identifiers	Title, authors, journal, publication year, county of publication, and sponsorship
Study design	Number of animals assigned to each experimental and control group and method of SNHL induction
Animal model characteristics	Animal species, gender, strain, age, method of SNHL induction, and immune status
Intervention characteristics	Source of MSCs, MSC identifiers including plastic adherence, positive/negative markers, differentiation, cell expansion media, passage number, cell dose, method of delivery, timing relative to SNHL induction, and frequency of MSC administration
Outcome measures	Method of assessing hearing outcome and timing of data collection relative to SNHL induction
Other	Long-term outcomes, type of control, MSC side effects (animal survival)

### Assessment of risk of bias

The risk of bias will be assessed independently by two individuals using the SYRCLE risk of bias tool [17]. The SYRCLE tool contains ten assessment domains related to selection bias, performance bias, detection bias, attrition bias, and reporting bias. For each included study, each domain will be scored as low, high, or unclear risk of bias.

### Assessment of external validity and construct validity

This systematic review will record characteristics that will assess external validity or the degree to which the results can be generalized to different experimental settings. This will assess the ability to replicate the study. This will be evaluated via subgroup analysis of the primary outcome based on species/strain of animal, control group, tissue source, route of administration, timing and dose of MSCs, and modifications to the MSCs. This data will assess the effects of factors such as animal characteristics and preparation/source of MSCs on the primary and secondary outcome. Furthermore, this will establish the ideal conditions for future preclinical and clinical trials.

Construct validity in preclinical research refers to the extent to which the animal study correlates to a clinical scenario [18]. Construct validity will be assessed according to how well the experimental design models SNHL in humans. The domains assessed will be animal subjects (small vs large animal), outcome measures of hearing assessment (whether they are the same ones used in humans), modeling of disease (noise induction vs medication induced), administration of intervention (clinically relevant such as intravenous, subcutaneous vs. non-clinically relevant, i.e., intraperitoneal). This will help determine whether the included studies enable generalization to a potential clinical study of stem cells for SNHL.

### Data analysis

Categorical variables will be presented as frequencies and percentages, and continuous variables will be pooled using the ratio of weighted means method with inverse variance random effects modeling [19]. When appropriate, dichotomous variables will be pooled and described as odds ratios and 95% CIs. Statistical heterogeneity of included studies will be measured with *I*^2^ tests with 95% uncertainty intervals [20]. If there is an adequate amount of studies, an assessment for the existence of publication bias will be conducted with funnel plot techniques and Egger’s regression test [21].

## Discussion

The knowledge gained from these studies is now impacting subsequent experiments that seek to assess the functional benefits of stem cell therapy as a treatment for SNHL. Current research has begun on early phases of clinical trials on the subject [11, 22]. The progression of research on SNHL in human subjects warrants further examination of the efficacy of mesenchymal stem cell (MSC) therapy in animal models. Improving methodology and standardizing the procedure in animal models could optimize results seen in human trials. While there are other studies that have examined the use of cell-free products (microvesicles, conditioned media, and miRNA) as a viable treatment, this systematic review focuses on the use of cell-based therapies, specifically MSCs.

This review will also focus on identifying various gaps or inconsistencies in preclinical experiments. By analyzing and comparing the current data on MSC treatment in animal models of SNHL, we intend to promote a more consistent method and directional approach.

There are several limitations to this study that are common across systematic reviews. The studies included will only be those published and will not include studies that are unpublished or not in English. Furthermore, it will be difficult to ascertain the clinical translatability of our findings, given that this study will only include preclinical animal models.

## Additional files

Additional file 1: Systematic Review Center for Laboratory animal Experimentation Protocol for. (PDF 459 kb)

Additional file 2: Search terms used in MEDLINE's PubMed. (DOCX 11 kb)

## Abbreviations

ABR: Auditory brainstem response
BAEPs: Brainstem auditory evoked potentials
CINAHL: Cumulative Index to Nursing and Allied Health Literature
DPOAE: Distortion product otoacoustic emissions testing
MSC: Mesenchymal stem/stromal cell
SNHL: Sensorineural hearing loss
SYRCLE: Systematic Review Centre for Laboratory Animal Experimentation
