# Supplementary material for: Mesenchymal stem cells for sensorineural hearing loss: protocol for a systematic review of preclinical studies
Source: Syst Rev. 2019 May 25;8:126. doi: 10.1186/s13643-019-1015-7 (PMC6535185; doi:10.1186/s13643-019-1015-7)
Supplement: Supplementary file 1 — Systematic Review Center for Laboratory animal Experimentation Protocol for. (PDF 459 kb) [file 13643_2019_1015_MOESM1_ESM.pdf]

SYSTEMATIC REVIEW PROTOCOL FOR ANIMAL INTERVENTION STUDIES

FORMAT BY SYRCLE (WWW.SYRCLE.NL)
VERSION 2.0 (DECEMBER 2014)

Item #	Section/Subsection/Item	Description	Check for approval
	A. General		
1.	Title of the review	Mesenchymal stem cells for sensorineural hearing loss: a systematic review of pre-clinical studies	
2.	Authors (names, affiliations, contributions)	Kevin Chorath: study design, data collection and analysis, manuscript writing<br>Nicolas Morton-Gonzaba: data collection and analysis, manuscript writing<br>Walter John Humann: study design<br>Matthew Willis: study design, data collection and analysis, manuscript writing<br>Alvaro Moreira: study design, statistical analysis, manuscript revising, supervision<br><br>University of Texas Health San Antonio<br>7703 Floyd Curl Dr., MC 7812<br>San Antonio, Texas, 78229 USA	
3.	Other contributors (names, affiliations, contributions)		
4.	Contact person + e-mail address	Alvaro Moreira: moreiraa@uthscsa.edu	
5.	Funding sources/sponsors	National Center for Advancing Translational Sciences, National Institutes of Health, through Grant KL2 TR001118.	
6.	Conflicts of interest	None	
7.	Date and location of protocol registration		
8.	Registration number (if applicable)		
9.	Stage of review at time of registration	Preliminary searches<br>Piloting study selection<br>Formal screening with final search criteria	
	B. Objectives		
	Background		
10.	What is already known about this disease/model/intervention? Why is it important to do this review?	Sensorineural hearing loss (SNHL) is the most common form of permanent hearing loss. Unfortunately, there is no proven therapy to cure SNHL. However, advances in regenerative medicine have shown mesenchymal stem cells are a novel therapy in improving hearing in animal models of SNHL. Despite promising findings, a methodical evaluation of preclinical studies has not been performed.	

		The purpose of this systematic review is to examine the potential use mesenchymal stem cells (MSC) as a therapy in animal models of SNHL.	
	Research question		
11.	Specify the disease/health problem of interest	Sensorineural hearing loss: congenital, age related, or induced	
12.	Specify the population/species studied	All animal species, all ages	
13.	Specify the intervention/exposure	Mesenchymal stem/stromal cells	
14.	Specify the control population	Sensorineural hearing loss with severity equivalent to experimental group, not receiving stem cell therapy	
15.	Specify the outcome measures	Primary outcome: Functional hearing assessment<br>Otoacoustic emissions (OAE)<br>Cochlear microphonic<br>Auditory brainstem response (ABR)<br>Electrocochleography<br>Summating potential<br>Tympanometry<br>Compound action potential<br>Brainstem auditory evoked potentials (BAEP)<br><br>Secondary outcome:<br>Imaging<br>Histology<br>Microscopy<br>Gene protein expression<br>Behavioral	
16.	State your research question (based on items 11-15)	Can MSCs improve sensorineural hearing loss in animals?	
	C. Methods		
	Search and study identification		
17.	Identify literature databases to search (*e.g.* Pubmed, Embase, Web of science)	X MEDLINE via PubMed ☐ Web of Science<br>X SCOPUS ☐EMBASE<br>X Other, namely: Science Direct, CINAHL, Google Scholar<br>☐Specific journal(s), namely:	
18.	Define electronic search strategies (*e.g.* use the step by step search guide[15] and animal search filters[20, 21])	When available, please add a supplementary file containing your search strategy: [insert file name]	
19.	Identify other sources for study identification	X Reference lists of included studies ☐Books<br>X Reference lists of relevant reviews<br>☐Conference proceedings, namely:<br>☐Contacting authors/ organisations, namely:<br>☐Other, namely:	
20.	Define search strategy for these other sources	Screening the reference lists for relevant titles and screening the abstracts of these relevant titles	
	Study selection		

21.	Define screening phases (*e.g.* pre-screening based on title/abstract, full text screening, both)	First phase screening based on title and abstract<br>Second phase full-text screening of the eligible articles	
22.	Specify (a) the number of reviewers per screening phase and (b) how discrepancies will be resolved	Two investigators (K. Chorath and M. Willis) will independently screen all the abstracts/full texts for the inclusion criteria. Differences of opinion in either phase that cannot be resolved by discussion will be resolved by consulting a third investigator (A. Moreira).	
	Define all inclusion and exclusion criteria based on:		
23.	Type of study (design)	<u>Inclusion criteria:</u><br>Animal intervention studies, regardless of the methodological quality<br><br><u>Exclusion criteria:</u><br>Non-intervention studies<br>No control group	
24.	Type of animals/population (*e.g.* age, gender, disease model)	<u>Inclusion criteria:</u><br>All genders<br>All ages<br><br><u>Exclusion criteria:</u><br>Humans<br>*In vitro*	
25.	Type of intervention (*e.g.* dosage, timing, frequency)		
26.	Outcome measures	<u>Primary outcome:</u> Functional hearing assessment<br><br><u>Secondary outcome:</u> Refer to objective 15	
27.	Language restrictions	Only English articles will be included	
28.	Publication date restrictions	None	
29.	Other		
30.	Sort and prioritize your exclusion criteria per selection phase	<u>Selection phase I:</u><br>1. Not a primary study<br>2. Not an *in vivo* animal study<br>3. Not SNHL<br>4. No MSC treatment<br><br><u>Selection phase II:</u><br>1. Not a primary study<br>2. Not an *in vivo* animal study<br>3. No SNHL<br>4. No MSC treatment<br>5. No control group	
	Study characteristics to be extracted (for assessment of external validity, reporting quality)		
31.	Study ID (*e.g.* authors, year)	Authors, journal, title, year, language, contact author e-	

		mail	
32.	Study design characteristics (*e.g.* experimental groups, number of animals)	Number of animals in experimental and control groups<br>Etiology for SNHL	
33.	Animal model characteristics (*e.g.* species, gender, disease induction)	Animal species, strain, age, gender, congenital, disease induction, immune status	
34.	Intervention characteristics (*e.g.* intervention, timing, duration)	Source, dose, route of delivery, timing, and frequency of MSCs	
35.	Outcome measures	Type and timing of outcome measures in paper	
36.	Other (*e.g.* drop-outs)	Reason of exclusion	
	Assessment risk of bias (internal validity) or study quality		
37.	Specify (a) the number of reviewers assessing the risk of bias/study quality in each study and (b) how discrepancies will be resolved	Two investigators (K. Chorath and M. Willis) will independently screen all the abstracts/full texts for the inclusion criteria. Differences of opinion in either phase that cannot be resolved by discussion will be resolved by consulting a third investigator (A. Moreira).	
38.	Define criteria to assess (a) the internal validity of included studies (*e.g.* selection, performance, detection and attrition bias) and/or (b) other study quality measures (*e.g.* reporting quality, power)	X By use of SYRCLE's Risk of Bias tool[4]<br>☐By use of SYRCLE’s Risk of Bias tool, adapted as follows:<br>☐By use of CAMARADES' study quality checklist, e.g [22]<br>☐By use of CAMARADES' study quality checklist, adapted as follows:<br>☐Other criteria, namely:	
	Collection of outcome data		
39.	For each outcome measure, define the type of data to be extracted (*e.g.* continuous/dichotomous, unit of measurement)	Primary/Secondary outcome: continuous data	
40.	Methods for data extraction/retrieval (*e.g.* first extraction from graphs using a digital screen ruler, then contacting authors)	Extraction from text, tables, and figures (GetData Graph Digitizer)	
41.	Specify (a) the number of reviewers extracting data and (b) how discrepancies will be resolved	Two investigators (K. Chorath and M. Willis) will independently screen all the abstracts/full texts for the inclusion criteria. Differences of opinion in either phase that cannot be resolved by discussion will be resolved by consulting a third investigator (A. Moreira).	
	Data analysis/synthesis		
42.	Specify (per outcome measure) how you are planning to combine/compare the data (*e.g.* descriptive summary, meta-analysis)		
43.	Specify (per outcome measure) how it will be decided whether a meta-analysis will be performed		
	If a meta-analysis seems feasible/sensible, specify (for each outcome measure):		
44.	The effect measure to be used (*e.g.* mean difference, standardized mean difference, risk ratio, odds ratio)	Continuous outcomes will be analysed using standardized mean differences (95% CI)	

45.	The statistical model of analysis (*e.g.* random or fixed effects model)	Random-effects model	
46.	The statistical methods to assess heterogeneity (*e.g.* I^2, Q)	I^2	
47.	Which study characteristics will be examined as potential source of heterogeneity (subgroup analysis)	Meta-regression analyses will be performed to examine heterogeneity on outcomes including: animal type, animal age, sex, species and strain, type of SNHL induction, type and tissue source of MSCs, timing, frequency, dosing of administration, route of cell administration, use of co-interventions	
48.	Any sensitivity analyses you propose to perform		
49.	Other details meta-analysis (*e.g.* correction for multiple testing, correction for multiple use of control group)		
50.	The method for assessment of publication bias	Funnel plots and Egger's test	

Final approval by (names, affiliations):
Kevin Chorath, Nicolas Morton-Gonzaba, Matthew Willis, Alvaro Moreira — Date: June 13, 2018
