# Supplementary material for: Mesenchymal stem cells for sensorineural hearing loss: protocol for a systematic review of preclinical studies
Source: Syst Rev. 2019 May 25;8:126. doi: 10.1186/s13643-019-1015-7 (PMC6535185; doi:10.1186/s13643-019-1015-7)
Supplement: Supplementary file 2 — Search terms used in MEDLINE's PubMed. (DOCX 11 kb) [file 13643_2019_1015_MOESM2_ESM.docx]

((((("mesenchymal stromal cells"[MeSH Terms] OR ("mesenchymal stromal cells"[MeSH Terms] OR ("mesenchymal"[All Fields] AND "stromal"[All Fields] AND "cells"[All Fields]) OR "mesenchymal stromal cells"[All Fields] OR ("mesenchymal"[All Fields] AND "stem"[All Fields] AND "cell"[All Fields]) OR "mesenchymal stem cell"[All Fields])) OR ("mesenchymal stromal cells"[MeSH Terms] OR ("mesenchymal"[All Fields] AND "stromal"[All Fields] AND "cells"[All Fields]) OR "mesenchymal stromal cells"[All Fields] OR ("mesenchymal"[All Fields] AND "stromal"[All Fields] AND "cell"[All Fields]) OR "mesenchymal stromal cell"[All Fields])) OR MSC[All Fields]) OR (regenerative[All Fields] AND ("cells"[MeSH Terms] OR "cells"[All Fields] OR "cell"[All Fields]))) AND ((((("hearing loss, sensorineural"[MeSH Terms] OR ("hearing loss, sensorineural"[MeSH Terms] OR ("hearing"[All Fields] AND "loss"[All Fields] AND "sensorineural"[All Fields]) OR "sensorineural hearing loss"[All Fields] OR ("sensorineural"[All Fields] AND "hearing"[All Fields] AND "loss"[All Fields]))) OR ("deafness"[MeSH Terms] OR "deafness"[All Fields])) OR ("retrocochlear diseases"[MeSH Terms] OR ("retrocochlear"[All Fields] AND "diseases"[All Fields]) OR "retrocochlear diseases"[All Fields] OR ("nerve"[All Fields] AND "deafness"[All Fields]) OR "nerve deafness"[All Fields])) OR ("hearing loss"[MeSH Terms] OR ("hearing"[All Fields] AND "loss"[All Fields]) OR "hearing loss"[All Fields] OR ("hearing"[All Fields] AND "impairment"[All Fields]) OR "hearing impairment"[All Fields])) OR ("hearing disorders"[MeSH Terms] OR ("hearing"[All Fields] AND "disorders"[All Fields]) OR "hearing disorders"[All Fields] OR ("hearing"[All Fields] AND "disorder"[All Fields]) OR "hearing disorder"[All Fields]))) AND ((preclinical[All Fields] OR experimental[All Fields]) OR ("animals"[MeSH Terms:noexp] OR animal[All Fields]))
